# Clinical trials on drug repositioning for COVID-19 treatment

**DOI:** 10.26633/RPSP.2020.40

**Published:** 2020-03-20

**Authors:** Sandro G. Viveiros Rosa, Wilson C. Santos

**Affiliations:** 1 Universidade Federal Fluminense Universidade Federal Fluminense Brazil Universidade Federal Fluminense, Rio de Janeiro, Brazil.

**Keywords:** Drug repositioning, clinical trials as topic, coronavirus infection, virus diseases, pneumonia, viral, pandemics, Reposicionamiento de medicamentos, ensayos clínicos como asunto, infecciones por coronavirus, virosis, neumonía viral, pandemias, Reposicionamento de medicamentos, ensaios clínicos como assunto, infecções por coronavirus, viroses, pneumonia viral, pandemias

## Abstract

The World Health Organization (WHO) was informed on December 2019 about a coronavirus pneumonia outbreak in Wuhan, Hubei province (China). Subsequently, on March 12, 2020, 125,048 cases and 4,614 deaths were reported. Coronavirus is an enveloped RNA virus, from the genus *Betacoronavirus*, that is distributed in birds, humans, and other mammals. WHO has named the novel coronavirus disease as COVID-19. More than 80 clinical trials have been launched to test coronavirus treatment, including some drug repurposing or repositioning for COVID-19. Hence, we performed a search in March 2020 of the clinicaltrials.gov database. The eligibility criteria for the retrieved studies were: contain a clinicaltrials.gov base identifier number; describe the number of participants and the period for the study; describe the participants’ clinical conditions; and utilize interventions with medicines already studied or approved for any other disease in patients infected with the novel coronavirus SARS-CoV-2 (2019-nCoV). It is essential to emphasize that this article only captured trials listed in the clinicaltrials.gov database. We identified 24 clinical trials, involving more than 20 medicines, such as human immunoglobulin, interferons, chloroquine, hydroxychloroquine, arbidol, remdesivir, favipiravir, lopinavir, ritonavir, oseltamivir, methylprednisolone, bevacizumab, and traditional Chinese medicines (TCM). Although drug repurposing has some limitations, repositioning clinical trials may represent an attractive strategy because they facilitate the discovery of new classes of medicines; they have lower costs and take less time to reach the market; and there are existing pharmaceutical supply chains for formulation and distribution.

## RESUMEN

En diciembre de 2019 fue informado a la Organización Mundial de la Salud (OMS) un brote de neumonía por coronavirus en Wuhan, provincia de Hubei, China. Al 12 de marzo de 2020, se habían notificado 125 048 casos y 4 614 muertes. El coronavirus es un virus ARN envuelto del género *Betacoronavirus* distribuido en aves, seres humanos y otros mamíferos. La OMS ha denominado a la nueva enfermedad por coronavirus COVID-19. Se han puesto en marcha más de 80 ensayos clínicos para evaluar un tratamiento para el coronavirus, que incluyen algunos ensayos de reposicionamiento de medicamentos para la COVID-19. En marzo de 2020 se llevó a cabo una búsqueda de los ensayos clínicos registrados en la base de datos clinicaltrials.gov. Los criterios de elegibilidad para los estudios recuperados fueron tener un número de identificación de la base de datos clinicaltrials.gov; describir el número de participantes y el período del estudio; describir las condiciones clínicas de los participantes; y emplear intervenciones con medicamentos ya estudiados o aprobados para cualquier otra enfermedad en pacientes infectados con el nuevo coronavirus SARS-CoV-2 (2019-nCoV). Es esencial destacar que este artículo solo recoge los ensayos que figuran en la base de datos clinicaltrials. gov. Se identificaron 24 ensayos clínicos relacionados con más de 20 medicamentos, como inmunoglobulina humana, interferones, cloroquina, hidroxicloroquina, arbidol, remdesivir, favipiravir, lopinavir, ritonavir, oseltamivir, metilprednisolona, bevacizumab y medicina tradicional china. Aunque el reposicionamiento de medicamentos tiene algunas limitaciones, el reposicionamiento de los ensayos clínicos puede representar una estrategia atractiva porque facilita el descubrimiento de nuevas clases de medicamentos; estos tienen costos más bajos y tardan menos en llegar al mercado; y existen cadenas de suministro farmacéutico que apoyan la formulación y la distribución.

## RESUMO

A Organização Mundial da Saúde (OMS) foi informada, em dezembro de 2019, sobre um surto de pneumonia por coronavírus em Wuhan, província de Hubei (China). Posteriormente, em 12 de março de 2020, 125 048 casos e 4 614 mortes haviam sido registrados. O coronavírus é um vírus RNA envelopado do gênero *Betacoronavírus,* distribuído em aves e em humanos e outros mamíferos. A OMS designou a nova doença por coronavírus como COVID-19. Mais de 80 ensaios clínicos foram iniciados para testar tratamentos para o coronavírus, incluindo alguns de reposicionamento de medicamentos para o COVID-19. Assim, em março de 2020 realizou-se uma busca na base de dados clinicaltrials.gov. Os critérios de elegibilidade para os estudos recuperados foram: conter o número identificador da base de dados clinicaltrials.gov; descrever o número de participantes e o período do estudo; descrever as condições clínicas dos participantes; e utilizar intervenções para tratamento de doentes infectados com o novo coronavírus SARS-CoV-2 (2019-nCoV) com medicamentos já estudados ou aprovados para qualquer outra doença. É essencial salientar que este artigo apenas capturou ensaios listados na base de dados clinicaltrials.gov. Foram identificados 24 ensaios clínicos envolvendo mais de 20 medicamentos, tais como imunoglobulina humana, interferons, cloroquina, hidroxicloroquina, arbidol, remdesivir, favipiravir, lopinavir, ritonavir, oseltamivir, metilprednisolona, bevacizumabe e medicamentos chineses tradicionais. Embora o reposicionamento de medicamentos tenha algumas limitações, os ensaios clínicos de reposicionamento podem representar uma estratégia atraente, porque facilitam a descoberta de novas classes de medicamentos, têm custos mais baixos, levam menos tempo para chegar ao mercado e se beneficiam de cadeias de fornecimento farmacêutico já existentes para formulação e distribuição.

The World Health Organization (WHO) was informed on December 31, 2019, about a pneumonia outbreak in Wuhan, Hubei province (China), a city with 11 million inhabitants. By March 12, 2020, there were 125 048 cases and 4 614 deaths (nearly 3.7% of cases) reported for the novel coronavirus (1), named 2019-novel coronavirus (2019-nCoV), and later renamed as severe acute respiratory syndrome coronavirus 2 (SARS-CoV-2) (2). The WHO named this novel coronavirus disease as COVID-19 (1,2), and there have been confirmed cases in 117 countries or territories outside China, including Japan, the United States of America, Italy, Iran, and Brazil (1). Coronavirus is an enveloped RNA virus, from the genus *Betacoronavirus*, that is distributed in birds, humans, and other mammals (3,4). New evidence indicates a link between SARS-CoV-2 and bat coronavirus (3). Six species of coronavirus are known as infectious in humans, four of which (229E, OC43, NL63, and HKU1) cause common cold symptoms (4). However, some authors have claimed that SARS-CoV-2 is even related to the coronavirus species responsible for the severe acute respiratory syndrome (SARS-CoV) as well as Middle East Respiratory Syndrome (MERS-CoV), which have zoonotic origins linked to severe significant illness with higher mortality (3,4). For example, in July 2003, the WHO reported 8 437 SARS-CoV cases, especially in China and Hong Kong, with 813 related deaths (5). Concerning MERS-CoV, from June 2012 to April 2018, 2 206 people were infected in 27 countries, 1 831 cases in Saudi Arabia, with 787 deaths (6). Unfortunately, there are no vaccines or medicines approved for the novel coronavirus infection (7), but more than 80 clinical trials have been launched to test coronavirus treatments, including some drug repurposing or repositioning for COVID-19 (8). Drug repositioning for other neglected diseases is an essential and universal strategy in the development of new drugs due to: *a)* lower costs and reduced time to reach the market because some clinical trial steps might not be required, especially concerning phases I and II; *b)* existing pharmaceutical supply chains are available for formulation and distribution; *c)* the possibility of combinations with other drugs in treatments that are more effective than monotherapy; and *d)* may facilitate the discovery of new mechanisms of action for old drugs and new classes of medicines (9,10).

On the other hand, this repurposing strategy has some limitations, including patent barriers, the complexity of regulatory pathways, absence of funding opportunities, greater access to data from other industry-sponsored clinical trials, and the heterogeneity of the population for new clinical studies (10). Nevertheless, drug repurposing is still a tool for the discovery of entirely new classes of medicines (10,11). Hence, considering this scenario, we felt that it is of interest to be aware of the drug repositioning in clinical tests for the COVID-2019 treatment.

## METHODS

We performed a search on March 12, 2020, at the clinicaltrials. gov database, with the descriptor [coronavirus] in the simple search field “conditions or disease” search, without restrictions on languages, disease conditions, results, or locations. The eligibility criteria for the retrieved studies were: contain a clinicaltrials.gov base identifier number; describe the number of participants and the study period; describe the patient’s clinical conditions; and interventions utilize medicines already studied or approved for any other disease in patients with COVID-19. ClinicalTrials.gov is a resource from the US National Library of Medicine, and it contains clinical studies conducted by 209 countries.

## RESULTS AND DISCUSSION

We identified 24 clinical trials (**Table 1**), in which 19 studies were at clinical phases 2, 3, or 4. The pharmaceutical interventions found for COVID-19 treatment include human immunoglobulin, interferons, chloroquine, hydroxychloroquine, arbidol, remdesivir, oseltamivir, favipiravir, carrimycin, methylprednisolone, bevacizumab, thalidomide, vitamin C, pirfenidone, bromhexine, fingolimod, danoprevir, ritonavir, darunavir, cobicistat, lopinavir, xiyanping, and traditional Chinese medicines (TCM).

**Chloroquine** and **hydroxychloroquine** are antimalarial drugs. They have antiviral effects against human immuno-deficiency virus (HIV), namely by inhibiting virus entry into host cells. Another antiviral mechanism is related to the post-translation alteration of newly synthesized proteins via glycosylation inhibition (12). Hydroxychloroquine is already being used in clinical trials on acquired immune deficiency syndrome (AIDS) treatment (13). In a recent trial with patients on COVID-19 treatment (14), 100% of patients treated with hydroxychloroquine in combination with the macrolide antibiotic **azithromycin** were virologically cured comparing with 57.1% in patients treated with hydroxychloroquine alone, and 12.5% in the control group. Currently, chloroquine and hydroxychloroquine will be tested (15,16) in patients with pneumonia caused by 2019-nCoV and chloroquine as preventative medicine for COVID-19, as shown in Table 1.

**Immunoglobulins** are useful in several diseases, such as idiopathic thrombocytopenia purpura (ITP), Guillain-Barre Syndrome (GBS), chronic inflammatory demyelinating polyneuropathy (CIDP), Kawasaki disease, and in multiple neurological autoimmune disorders refractory to standard immunosuppressive treatments (17). Broadly neutralizing antibodies can recognize a wide variety of glycoproteins (GPs) in virus surfaces or the protein shell of a non-enveloped virus. However, HIV-1, dengue virus (DENV), influenza viruses, hepatitis C virus (HCV), and Ebola virus (EBOV) can mutate superficial GPs in order to evade the antibody response, an obstacle in the development of new therapies against such infections (18). Trial NCT04261426 (19) is utilizing human immunoglobulin in patients with pneumonia caused by 2019-nCoV (Table 1).

Two clinical studies refer to the use of **remdesivir** in severe (20) or mild (21) respiratory infections by SARS-CoV-2. Remdesivir is a nucleotide analog inhibitor of the EBOV RNA-polymerase RNA-dependent (RdRp). Dyer et al. 2019 (22) described preliminary findings of a mortality rate of 33% in 499 patients treated with remdesivir against the EBOV disease in early infection stages. The same authors noted a mortality rate of 75% (almost 1 900 people) of non-treated infected patients during the same epidemic period (22). Wang et al. 2020 (23) presented data showing that remdesivir is effective against the 2019-nCoV in Vero E6 cells (EC_90_ 1.76 μM). The suggested mechanism for remdesivir involves the host cells’ post-entry stage (23).

**Arbidol**, also known as **umifenovir,** is approved in Russia and China for the treatment of influenza virus infections; it does not have significant adverse effects and is patented for SARS treatment (24). As shown in Table 1, four clinical trials will be conducted for COVID-19 treatment: one with arbidol in comparison with the basic treatment (25), and the other three studies comparing effects with oseltamivir (26,27), lopinavir-ritonavir (27), and carrimycin (28). The arbidol anti-viral mechanism against influenza A and B involves viral fusion inhibition with the targeted membrane, which blocks virus entry into the cell (24). **Oseltamivir** is another drug approved for influenza A and B treatment; it inhibits the viral neuraminidase and, consequently, blocks the release of viral particles from host cells, reducing the spread in the respiratory tract (29). Additionally, the use of oseltamivir was already reported during the COVID-19 epidemic in China, either with or without antibiotics and corticosteroids (30). Oseltamivir is also used in a clinical trial with multiple combinations with chloroquine and **favipiravir** (31), a nucleoside analog that is well-known as a broad-spectrum antiviral drug; it has shown (23) an EC_50_ of 61.88 μM against SARS-CoV-2 and low toxicity (CC50 >400 μM).

The **lopinavir-ritonavir** combination is approved for AIDS treatment in several countries. Both drugs are HIV protease inhibitors, but ritonavir is also a cytochrome P450 and GP inhibitor, a fact that endorses the lopinavir pharmacokinetic and pharmacodynamic activities against HIV (32). Such a combination, plus β-1b interferon, is in phase 2 for the MERS treatment (33). Several trials involve lopinavir-ritonavir treatment in comparison with the use of other drugs for COVID-19: arbidol (26,27), carrimycin (28), TCM (34,35), xiyanping (36,37), danoprevir-ritonavir (38) and interferon inhalation (34,38). Nevertheless, one previous article argued that in a clinical trial with 199 patients with laboratory-confirmed SARS-CoV-2 infection, the lopinavir-ritonavir combination was not associated with clinical improvement comparing with standard care procedures (39).

**TABLE 1.** Clinical trials identified at Clinicaltrials.gov related to drug repositioning for COVID-19 treatment

Intervention	Clinical condition	Sponsor	N° test / Status	Beginning / Estimated end	Phase
**Hydroxychloroquine**	30 participants with pneumonia caused by 2019-nCoV	Shanghai Public Health Clinical Center	NCT04261517 / Recruiting patients	6-2-2020 / 31-12-2020	3
**Chloroquine**	10000 participants in a prophylaxis study for COVID-19	University of Oxford	NCT04303507 / Not yet recruiting	May 2020 / May 2022	N/A
**Human immunoglobulin**	Pneumonia caused by 2019-nCoV with 80 participants	Peking Union Medical College Hospital	NCT04261426 / Not yet recruiting patients	10-2-2020 / 30-06-2020	2 and 3
**Remdesivir**	Severe respiratory infection caused by 2019-nCoV with 452 participants	Capital Medical University	NCT04257656 / Recruiting patients	6-2-2020 / 31-05-2020	3
**Remdesivir**	308 participants with mild/ moderate respiratory infection caused by 2019-nCoV	Capital Medical University	NCT04252664 / Recruiting patients	05-02-2020 / 27-04-2020	3
**Arbidol (umifenovir)**	Pneumonia caused by 2019-nCoV with 380 participants	Jieming QU, Ruijin Hospital	NCT04260594 / Not yet recruiting patients	7-02-2020 / 30-12-2020	4
**Arbidol** or **lopinavir-ritonavir** or **oseltamivir**	400 participants infected with 2019-nCoV	Tongji Hospital	NCT04255017 / Recruiting patients	01-02-2020 / 01-07-2020	4
**Arbidol** or **lopinavir-ritonavir**	125 participants infected with 2019-nCoV	Guangzhou 8th People’s Hospital	NCT04252885 / Recruiting patients.	28-01-2020 / 31-07-2020	4
**Darunavir-cobicistat** combination	Pneumonia caused by 2019-nCoV with 30 participants	Shanghai Public Health Clinical Center	NCT04252274 / Recruiting patients	30-01-2020 / 31-12-2020	3
**TCM** combination with **lopinavir-ritonavir, a-interferon** via aerosol	150 participants infected with 2019-nCoV	Beijing 302 Hospital	NCT04251871 / Recruiting patients	22-01-2020 / 22-01-2021	N/A
**Recombinant human interferon α2β**	328 participants with COVID-19	Tongji Hospital	NCT04293887 / Not yet recruiting	01-03-2020 / 30-06-2020	1
**Carrimycin** or **lopinavir-ritonavir** or **arbidol** or **chloroquine phosphate**	520 participants with COVID-19	Beijing YouAn Hospital	NCT04286503 / Not yet recruiting	23-02-2020 / 28/02-2021	4
**Danoprevir-ritonavir** and **interferon** inhalation or **lopinavir-ritonavir** or **TCM** plus **interferon** inhalation	50 participants with pneumonia caused by 2019-nCoV	The Ninth Hospital of Nanchang	NCT04291729 / Recruiting	14-02-2020 / 30-04-2020	4
**Xiyanping** or **lopinavir-ritonavir-interferon** inhalation	384 participants with pneumonia caused by 2019-nCoV	Jiangxi Qingfeng Pharmaceutical Co. Ltd.	NCT04275388 / Not yet recruiting	19-02-2020 / 14-12-2020	N/A
**Xiyanping** combined with **lopinavir-ritonavir**	80 participants with COVID-19	Jiangxi Qingfeng Pharmaceutical	NCT04295551 / Not yet recruiting	14-03-2020 / 14-04-2021	N/A
Combinations of **oseltamivir, favipiravir,** and **chloroquine**	80 participants with COVID-19	Rajavithi Hospital	NCT04303299 / Not yet recruiting	15-03-2020 / 30-11-2020	3
**Thalidomide**	40 participants with COVID-19	First Affiliated Hospital of Wenzhou Medical University	NCT04273581 / Not yet recruiting	18-02-2020 / 30-05-2020	2
**Thalidomide**	100 participants with pneumonia caused by 2019-nCoV	First Affiliated Hospital of Wenzhou Medical University	NCT04273529 / Not yet recruiting	20-02-2020 / 30-06-2020	2
**Vitamin C**	140 participants with severe pneumonia caused by 2019-nCoV	ZhiYong Peng	NCT04264533 / Recruiting	14-02-2020 / 30-09-2020	2
**Methylprednisolone**	80 participants infected with 2019-nCoV	Peking Union Medical College Hospital	NCT04244591 / Recruiting patients	26-01-2020 / 25-12-2020	2
**Pirfenidone**	294 participants with severe pneumonia caused by 2019-nCoV	Huilan Zhang	NCT04282902 / Recruiting	04-02-2020 / 01-06-2020	3
**Bromhexine hydrochloride**	60 participants with suspected and mild pneumonia caused by 2019-nCoV	Second Affiliated Hospital of Wenzhou Medical University	NCT04273763 / Enrolling by invitation	16-02-2020 / 30-04-2020	N/A
**Bevacizumab**	20 participants with severe COVID-19 pneumonia	Qilu Hospital of Shandong University	NCT04275414 / Recruiting	February 2020 / May 2020	2 and 3
**Fingolimod**	30 participants with COVID-19	1° Affiliated Hospital of Wenzhou Medical University	NCT04280588 / Recruiting	22-02-2020 / 01-06-2020	2

COVID-19, coronavirus disease 2019; 2019-nCoV, novel coronavirus 2019; TCM, traditional Chinese medicine

**Carrimycin** is a macrolide antibiotic with effects against some gram-positive bacteria and *in vitro* effects on *Mycobacterium tuberculosis* (40).

**Danoprevir** is an HCV NS3 protease inhibitor approved in China for the treatment of non-cirrhotic genotype 1b chronic hepatitis C, in combination with ritonavir, peginterferon-a, and ribavirin (41).

**Traditional Chinese medicine** (TCM) uses phytotherapeutic formulations such as teas, pills, powders or tinctures, and cultural components that originated 5000 years ago in Chinese medicine (42). TCMs were already used for SARS-CoV infection in 2002 as coadjuvant therapy with the enhancement of patients’ symptoms, increased oxyhemoglobin arterial saturation; they proved useful in the early stages of this infection (42).

**Interferons (IFNs)** are proteins that bind to cellular surfaces’ receptors and initiate JAK-STAT signaling cascades, with transcriptional regulation of genes controlled by interferons and effects against some viruses like hepatitis B virus and HCV (43).

**Xiyanping** is a TCM preparation with andrographolide as a principal component; it has significant antibacterial and antiviral effects (44).

**Darunavir,** in combination with **cobicistat,** will be used in trial number NCT04252274 (45) in patients with COVID-19 pneumonia. The United States Food and Drug Administration (FDA) currently approves such a combination in AIDS treatment. Darunavir is another HIV protease inhibitor, and cobicistat, like ritonavir, is a booster for enhancing the pharmacokinetics and pharmacodynamics of darunavir by cytochrome P450 (CYP3A) inhibition (46,47).

**Recombinant human interferon α2β** is described to have inhibitory effects on MERS-CoV and SARS-CoV (48), and the purpose of the clinical trials found for this paper is to evaluate the efficacy and safety of recombinant human interferon **α2β** in treating patients with new coronavirus infection (49).

**Thalidomide** will be used in two trials against COVID-19 (49, 50). Thalidomide has an anti-inflammatory action due to its ability to speed up the degradation of messenger RNA in blood cells and thus reduce tumor necrosis factor-α (TNFα). Furthermore, thalidomide can increase the secretion of interleukins, such as IL-12, and activate natural killer cells (51).

The corticosteroid **methylprednisolone** will be tested against COVID-19 (52). Long et al. 2016 (53) reported that corticosteroid therapy (methylprednisolone, dexamethasone, and hydrocortisone) is beneficial in treating SARS-CoV patients; it significantly prolongs the survival time of clinical cases. Nevertheless, other authors described the use of corticosteroids in the early stages of SARS infection with increasing values of viral load (54). Furthermore, studies with corticosteroids in the adjuvant therapy of MERS-CoV infection were unable to prove efficacy because all patients died (55). Methylprednisolone has already been used in COVID-19 patients in combination with antibiotics, oseltamivir, and oxygen therapy (56).

Finally, vitamin C (ascorbic acid), pirfenidone, bevacizumab, fingolimod, and bromhexine hydrochloride are going to be tested on COVID-19 (57-61). **Vitamin C** has antioxidant activity and may reduce oxidative stress and inflammation (57,62), effects that improve vasopressor synthesis, enhance immune cell function, improve endovascular function, and provide epigenetic immunologic modifications. Clinical trials have demonstrated promising data on mortality improvement in sepsis, but more extensive studies are necessary to validate these conclusions (63). **Pirfenidone** has been used in the treatment of idiopathic pulmonary fibrosis diseases due to anti-inflammatory and anti-oxidant effects, namely by inhibiting IL-1β and IL-4 (58). Trial NCT04282902 claimed (58) that anti-inflammatory effects may be helpful in SARS-CoV-2 infection. **Bevacizumab** is a humanized monoclonal antibody that targets vascular endothelial growth factor (VEGF) (59,63), and it may reduce the levels of VEGF caused by hypoxia, severe inflammation, and upregulation of the infected respiratory tract epithelium, all of which might suppress the edema in patients with COVID-19 (63). **Fingolimod** is a sphingosine-1-phosphate receptor regulator (FTY720) with an effective immunology modulator that is useful in multiple sclerosis (60). According to some pathological findings of pulmonary edema and hyaline membrane formation, the use of immune modulators, together with ventilator support, should be considered for severe patients to prevent the development of acute respiratory distress syndrome (ARDS). Study NCT04280588 aims to determine the efficacy of fingolimod for COVID-19 (60). **Bromhexine** is a transmembrane protease serine inhibitor; such a protease is responsible for the activation of S-glycoprotein of SARS-CoV and MERS-CoV for viral entry through the plasma membrane (61,64). One study (60) will evaluate the efficacy of bromhexine combined with standard treatment/standard treatment in patients with COVID-19.

In conclusion, the WHO declared an epidemic of pneumonia caused by the SARS-CoV-2 in 2020. In this review, we found 24 clinical trials that have already started with the repositioning of more than 20 medicines for COVID-19 treatment, such as human immunoglobulin, interferons, chloroquine, hydroxychloroquine, arbidol, remdesivir, favipiravir, oseltamivir, thalidomide, methylprednisolone, bevacizumab, and TCM. The Hydroxychloroquine-azithromycin combination was the first drug repurposed with excellent results in clinical trials against SARS-CoV-2, but further, more extended studies, with a higher number of patients, are needed to confirm these results. Besides its limitations, repositioning clinical trials are still an attractive strategy: they may facilitate the discovery of new classes of medicines; they may reduce the costs and time to reach the market; there is an existing pharmaceutical supply chain for formulation and distribution; and there is the possibility of combinations with other drugs in treatments that are more effective than monotherapy. Most of the studies found in this article are scheduled to end in 2020, and we hope these repositioning trials may help to find solutions for COVID-19 treatment by this year.

## Disclaimer.

Authors hold sole responsibility for the views expressed in the manuscript, which may not necessarily reflect the opinion or policy of the RPSP/PAJPH and/or PAHO.
